# Supplementary material for: BMI1 regulates PRC1 architecture and activity through homo- and hetero-oligomerization
Source: Nat Commun. 2016 Nov 9;7:13343. doi: 10.1038/ncomms13343 (PMC5105191; doi:10.1038/ncomms13343)
Supplement: Supplementary Information — Supplementary Figures 1-14. [file ncomms13343-s1.pdf]

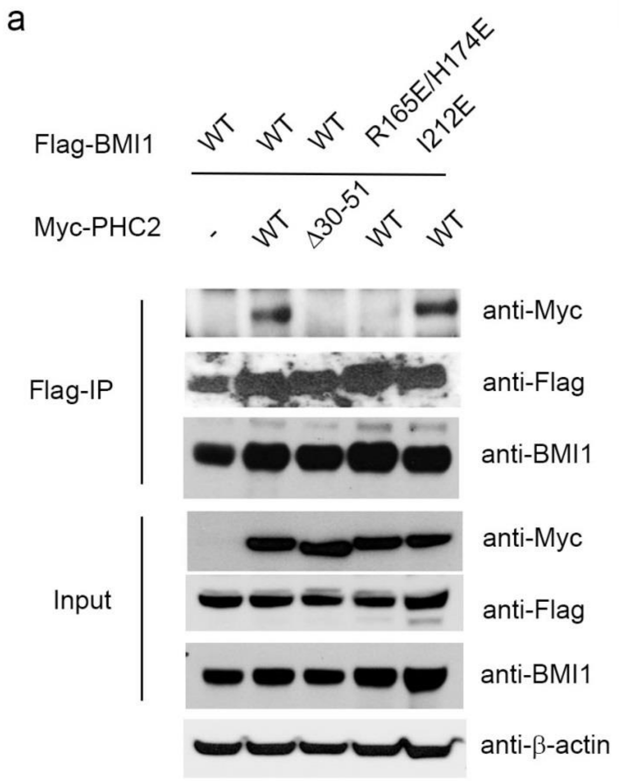


**Supplementary Figure 1**. (**a**) Flag-tag co-immunoprecipitations from HEK293 cells transfected with Flag-tagged full length BMI1 wild-type or mutants (R165E/H174E or I212E) and Myc-tagged PHC2_B or Myc-PHC2_B with deleted residues 30-51 (PHC2Δ30-51). Western blots are probed as indicated. (**b**) Flag-tag co-immunoprecipitation from HEK293 cells transfected with Flag-tagged full length BMI1 wild-type or BMI1 106-326. 500μg whole cell lysates were used for immunoprecipitation experiments. Western blots are probed as indicated.

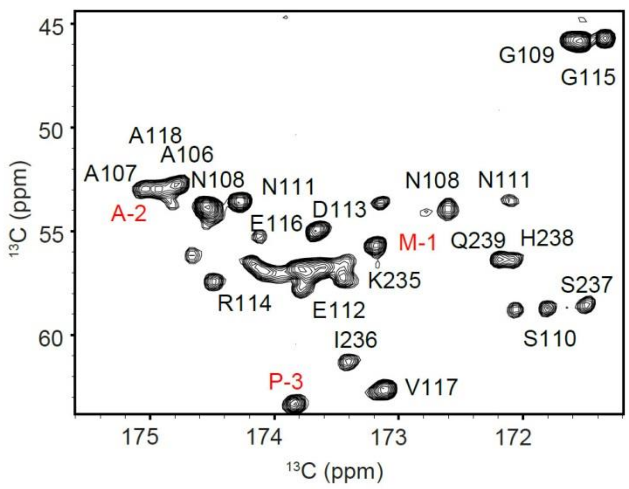


**Supplementary Figure 2:** $^{13}$C-detected 2D CBCACO spectrum for BMI1$_{106\text{-}240}$ with assignment for flexible regions. Residues remaining from tag cleavage during purification are colored in red.

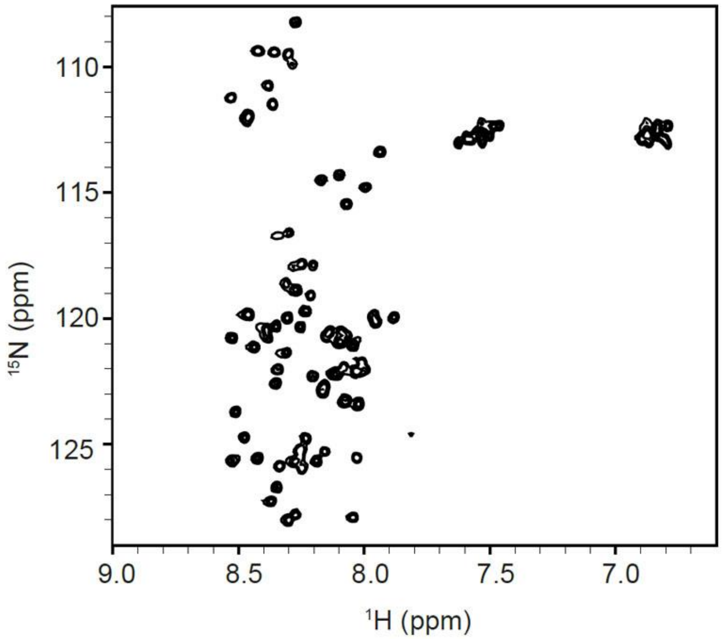


**Supplementary Figure 3.** $^{1}$H-$^{15}$N HSQC spectrum of 50 μM PHC2$_{1-79}$.

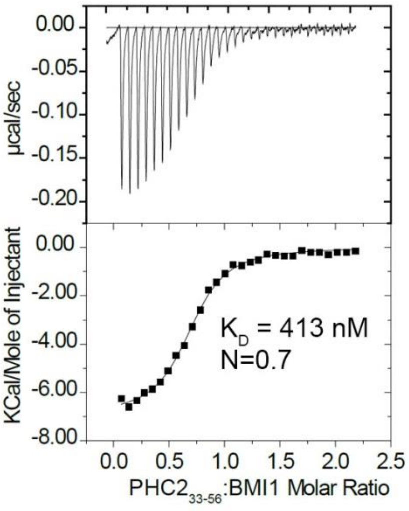


**Supplementary Figure 4.** Isothermal titration calorimetry experiments for BMI1 UBL titrated with $PHC2_{33-56}$ peptide.

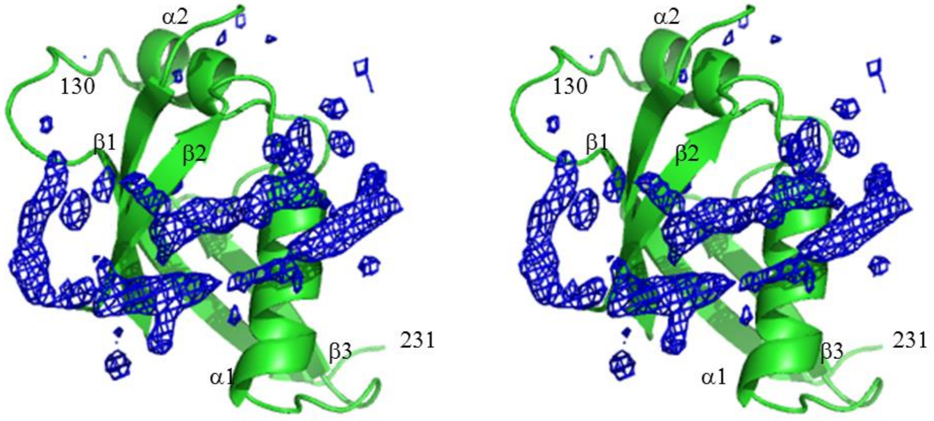


**Supplementary Fig 5**. Stereo view of the Fo - Fc omit electron density maps contoured at 3σ level.

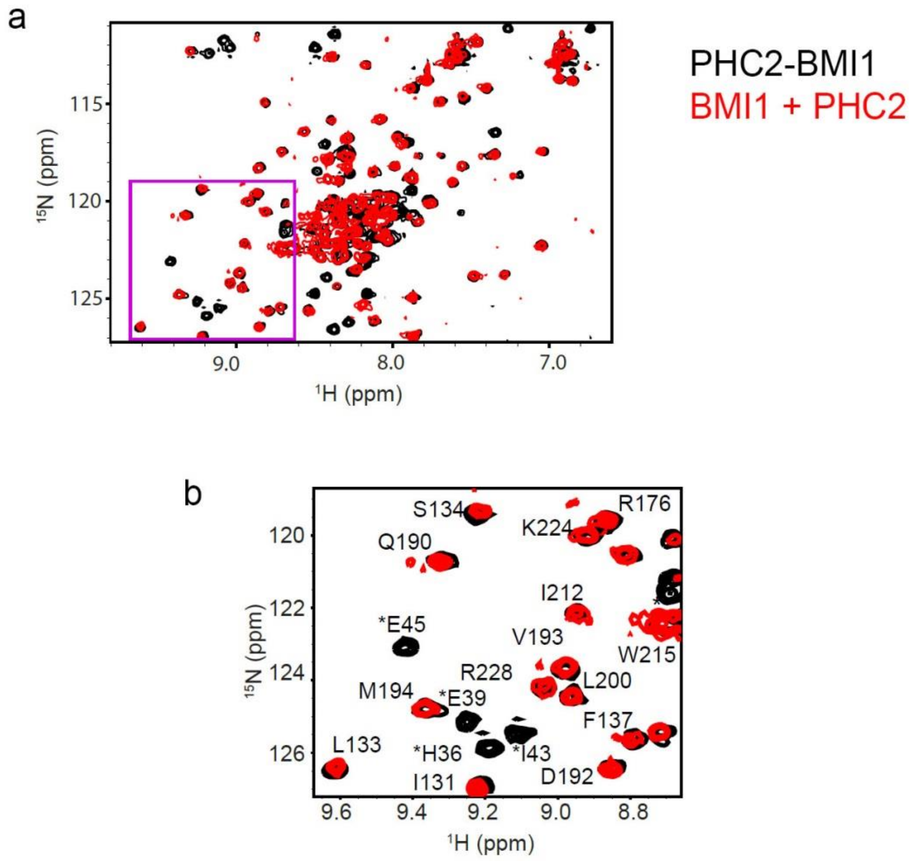


**Supplementary Figure 6**. (**a**) Superposition of $^{1}$H-$^{15}$N HSQC spectra for 50 μM $^{15}$N PHC2$_{30\text{-}64}$-BMI1 fusion protein (black) and 60 μM $^{15}$N BMI1 UBL saturated with 60 μM PHC2$_{32\text{-}61}$ (red). (**b**) Fragment of same spectra outlined in purple box with assignment. Residues from PHC2 fragment of fusion protein are indicated with asterisk.

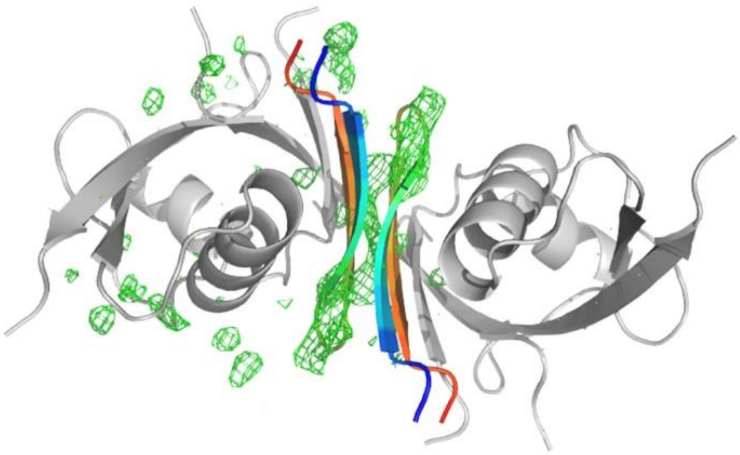

**Supplementary Figure 7.** Superposition of the structure of PHC2-BMI1 with the Fo-Fc difference omit map at 3 σ level (green) for the crystal structure of BMI1 UBL obtained in the presence of PHC2 peptide. PHC2 is colored from blue at the N-terminus to red at the C-terminus.

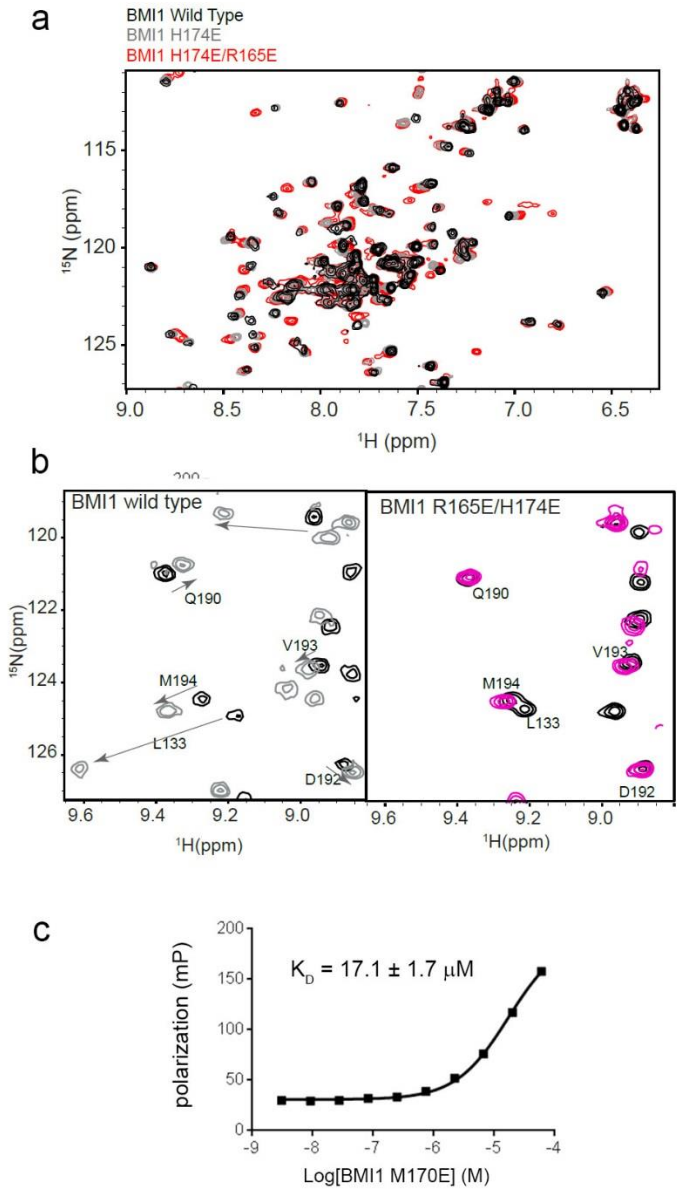


**Supplementary Figure 8.** (**a**) Comparison of $^{1}H$-$^{15}N$ HSQC spectra for 80 µM BMI1 UBL wild-type (black), 50 µM BMI1 UBL H174E (gray) and 100 µM BMI1 UBL R165E/H174E (red). (**b**) Fragment of $^{1}H$-$^{15}N$ HSQC spectra for 60 µM BMI1 UBL-domain wild-type (left) or 100 µM R165E/H174E (right) in the absence (black) or presence of $PHC2_{33\text{-}56}$ at 1-1 molar ratio (gray and magenta, respectively). (**c**) Binding affinity of PHC2 with BMI1 M170E mutant determined using fluorescence polarization (FP) experiments.

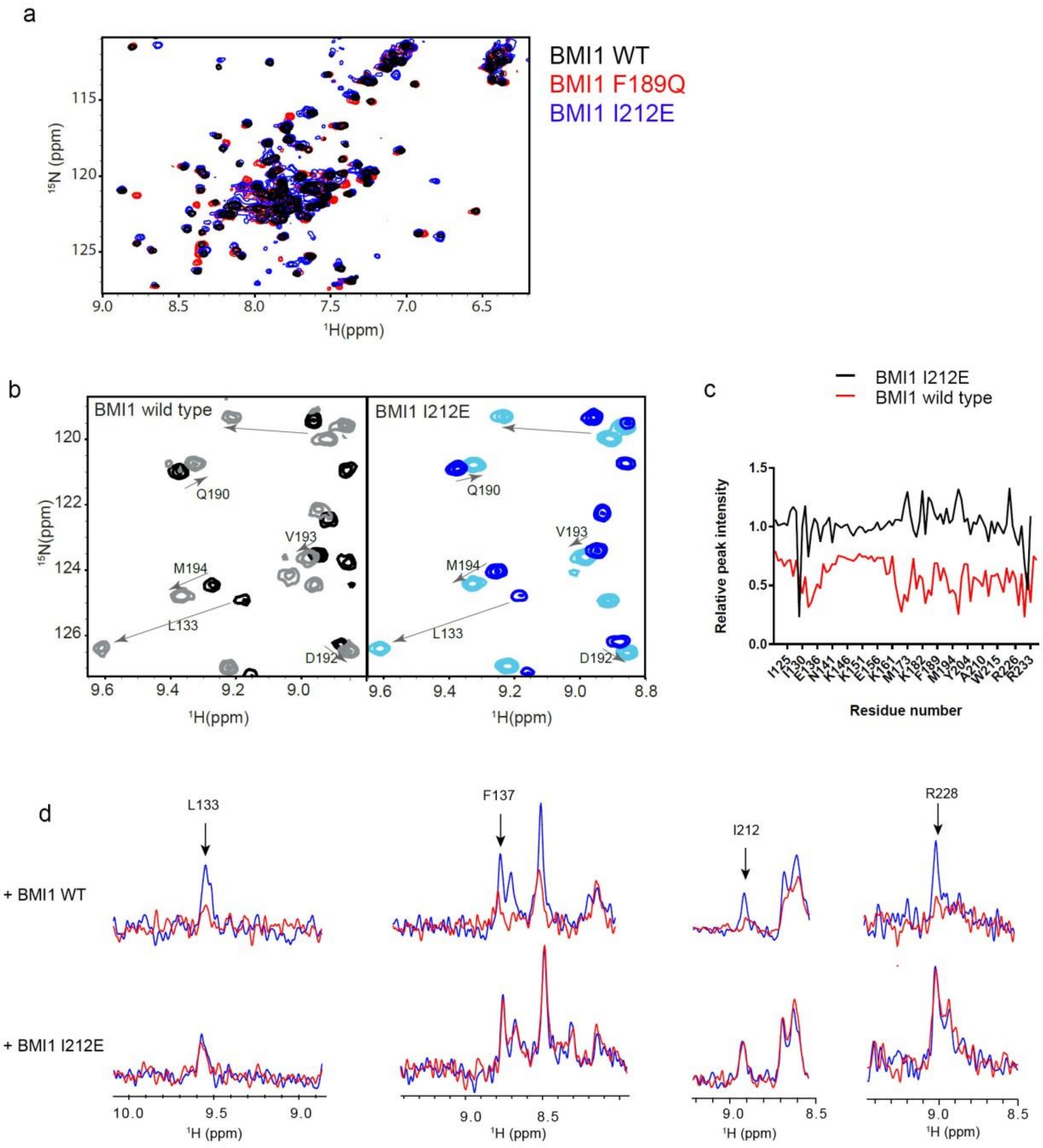


**Supplementary Figure 9**. (**a**) Comparison of $^{1}$H-$^{15}$N HSQC spectra for 80 µM BMI1 UBL wild-type (black), 100 µM BMI1 UBL F189Q (red) and 100 µM BMI1 UBL I212E (blue). (**b**) Fragments of $^{1}$H-$^{15}$N HSQC spectra for 60 µM wild-type BMI1 UBL (black, left) and 100 µM BMI1 UBL I212E (dark blue, right) superimposed with the spectra for BMI1 UBL with PHC2$_{33\text{-}56}$ peptide at 1-1 molar ratio (gray and light blue, respectively). Resonances perturbed by PHC2 binding are labeled and arrows indicate direction of chemical shift perturbation. (**c**) Quantification of peak intensities from $^{1}$H-$^{15}$N HSQC spectra for 50 µM $^{15}$N BMI1 UBL-PHC2$_{33\text{-}56}$ complex mixed with 100 µM unlabeled BMI1 UBL I212E-PHC2$_{33\text{-}56}$ complex (black) or 100 µM unlabeled BMI1 UBL wild-type-PHC2$_{33\text{-}56}$ complex (red). Intensities are normalized to 50 µM $^{15}$N BMI1 UBL -PHC2$_{33\text{-}56}$ complex. (**d**) Cross-sections for selected peaks from spectra in Figures 3E and 3F. Top row: selected peaks from $^{1}$H-$^{15}$N HSQC spectrum for 50 µM $^{15}$N BMI1 UBL-PHC2$_{33\text{-}56}$ complex (blue) mixed with 100 µM unlabeled wild-type-PHC2$_{33\text{-}56}$ complex (red).

Bottom row: selected peaks from $^{1}H$-$^{15}N$ HSQC spectrum for 50 µM $^{15}N$ BMI1 UBL-$PHC2_{33\text{-}56}$ complex (blue) mixed with 100 µM BMI1 UBL I212E-$PHC2_{33\text{-}56}$ complex (red).

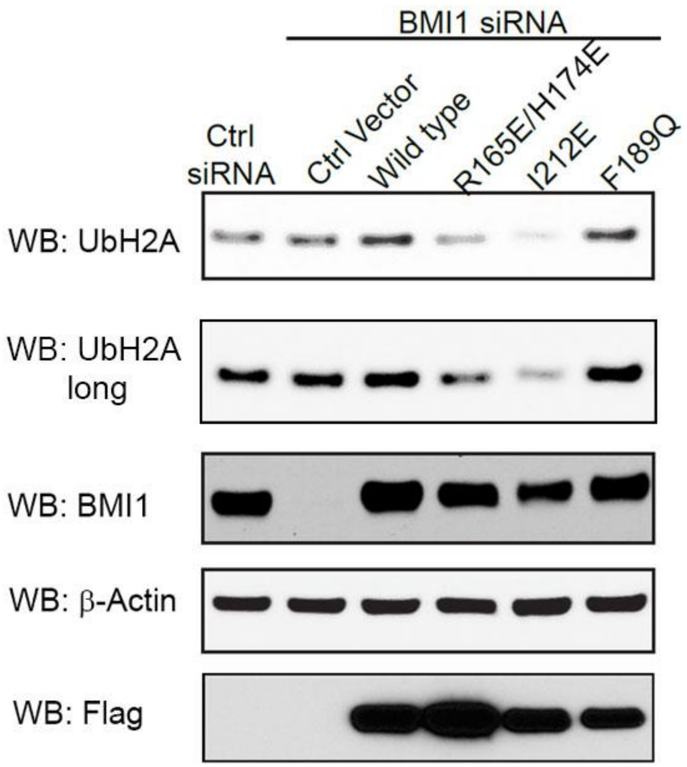


**Supplementary Figure 10**. Characterization of Ub-H2A levels upon overexpression of BMI1 mutants in HeLa cells. HeLa cells transfected with control or BMI1 3' UTR siRNA for 48 hours were transfected with plasmids encoding Flag-tagged full length wild-type BMI1 or mutants and analyzed by immunoblotting after 48 hours. UbH2A long represents longer exposure.

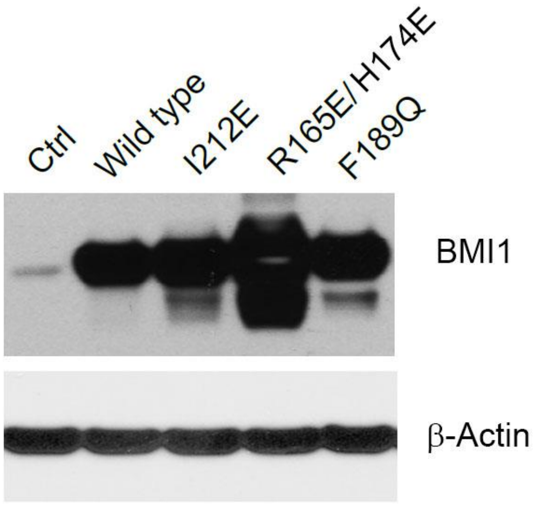


**Supplementary Figure 11**. Analysis of BMI1 expression in whole cell lysate of U2OS cells used for clonogenic survival assays expressing BMI1 shRNA and transfected with BMI1 wild-type or mutants. Western blot probed as indicated.

a

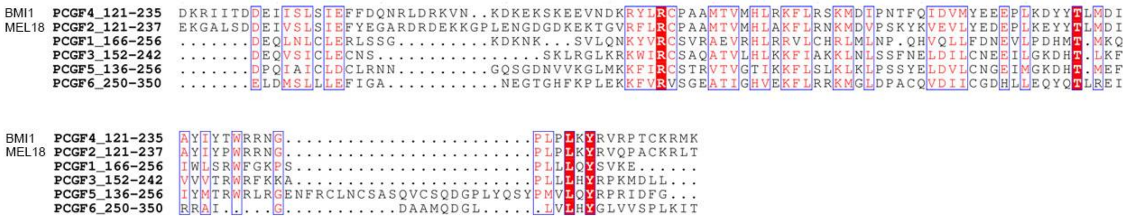


b

| Sequence identity | PCGF2 (MEL18) | PCGF3 | PCGF4 (BMI1) | PCGF5 | PCGF6 |
|---|---|---|---|---|---|
| PCGF1 | 24.44 | 38.10 | 31.11 | 28.57 | 23.17 |
| PCGF2 | | 30.00 | 60.00 | 28.57 | 30.11 |
| PCGF3 | | | 31.11 | 50.55 | 28.57 |
| PCGF4 | | | | 27.84 | 26.88 |
| PCGF5 | | | | | 27.84 |

c

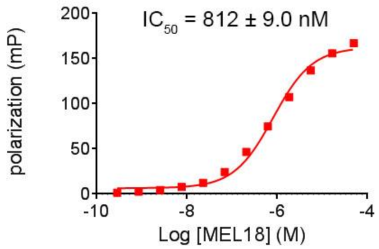


**Supplementary Figure 12**. (**a**) Sequence alignment of UBL domains for PCGF family members. (**b**) Matrix showing sequence identity for UBL domains among PCGF family members. UBL domain of BMI1 (PCGF4) shares 60% sequence identity with MEL18 (PCGF2). (**c**) Fluorescence polarization experiment showing the affinity of $MEL18_{121-237}$ binding with $PHC2_{32-61}$.

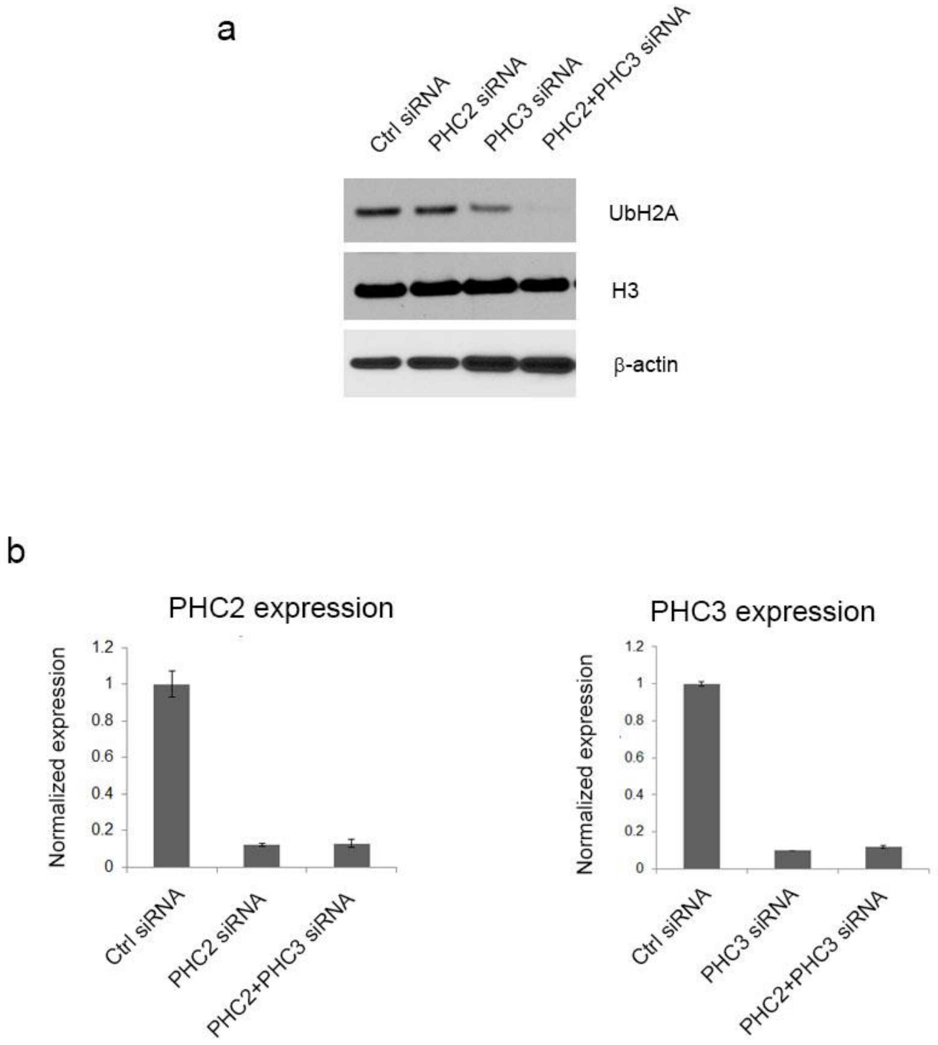


**Supplementary Figure 13**. (**a**) Analysis of the Ub-H2A levels upon knockdown of PHC2 and PHC3 in HeLa cells. (**b**) Expression of PHC2 and PHC3 in HeLa cells upon siRNA knockdown assessed by RT-qPCR.

**Figure 1b**

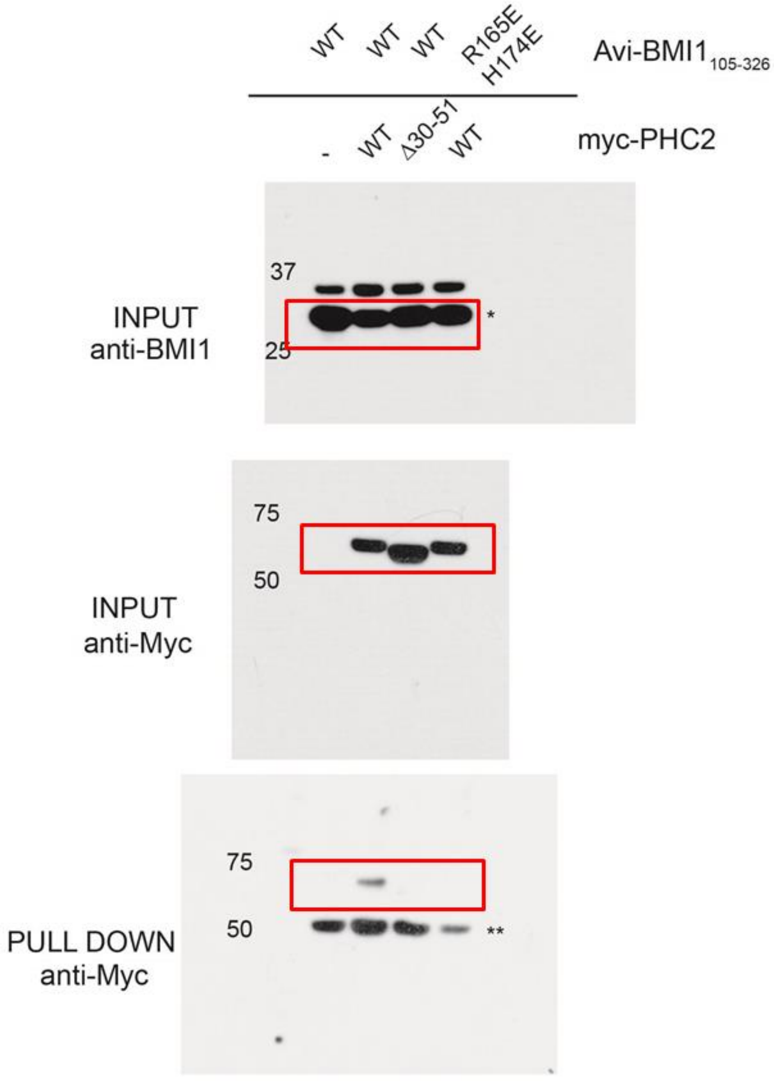


* Endogenous BMI
** myc non-specific

**Figure 4a**

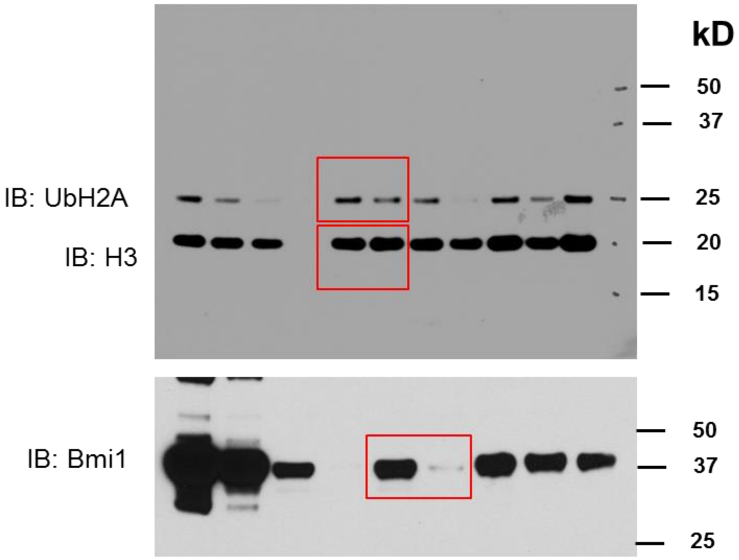


**Figure 4b and Supplementary Fig 10**

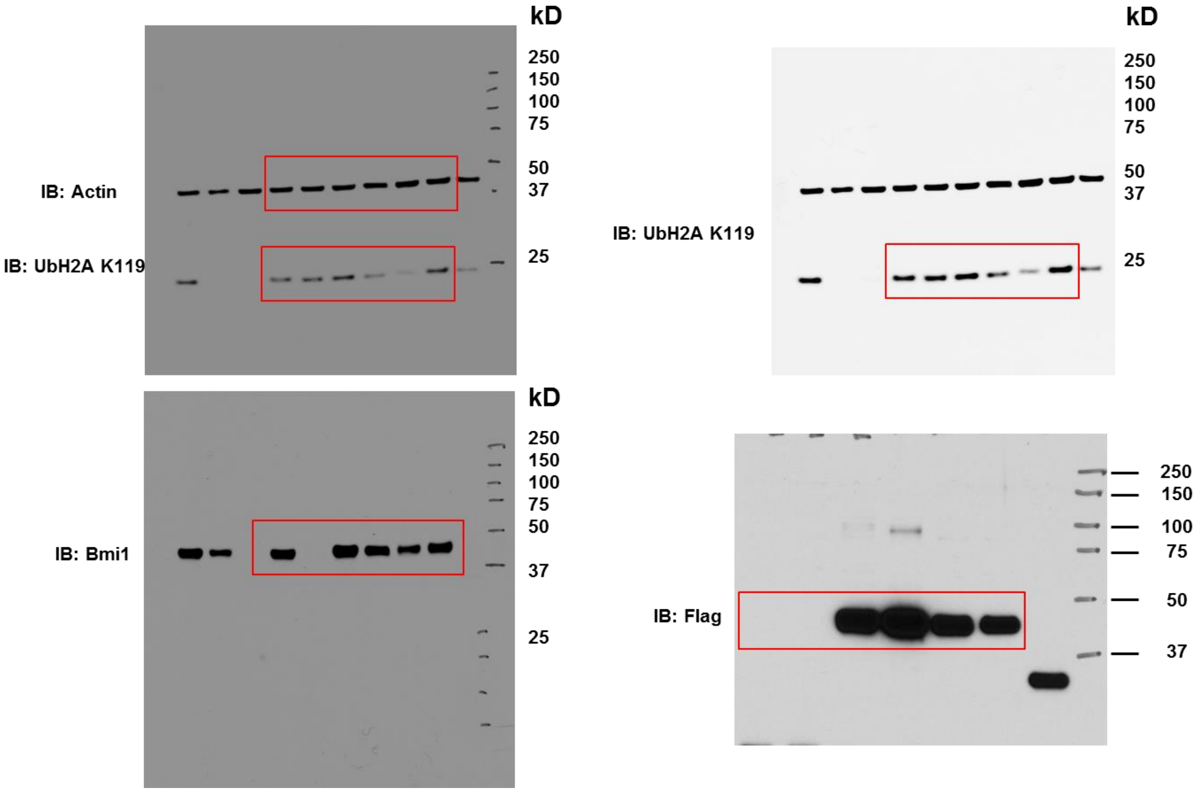

**Figure 4c**

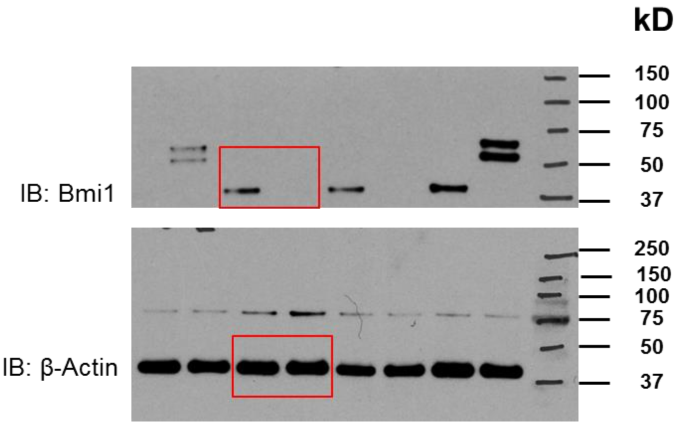


**Supplementary Fig 1a**

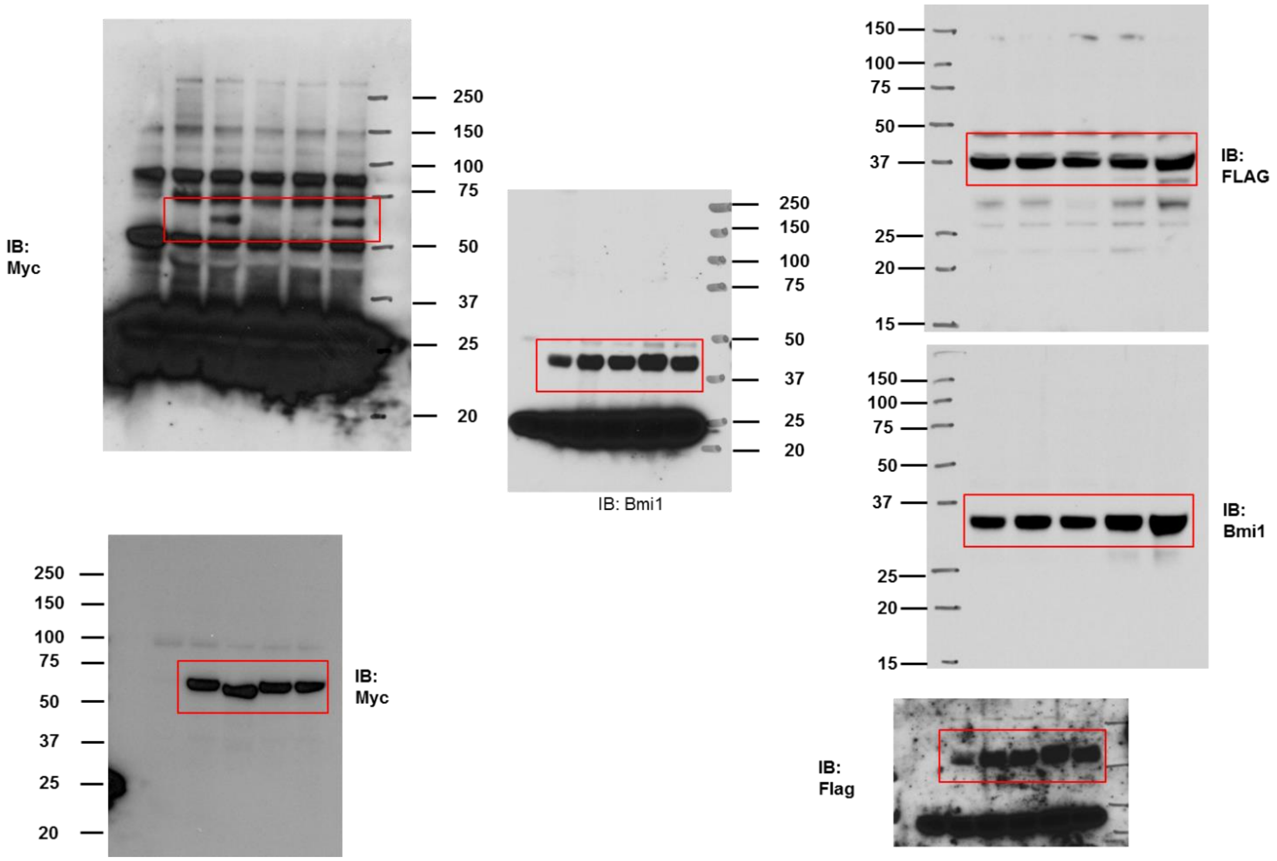

## Supplementary Fig 1b

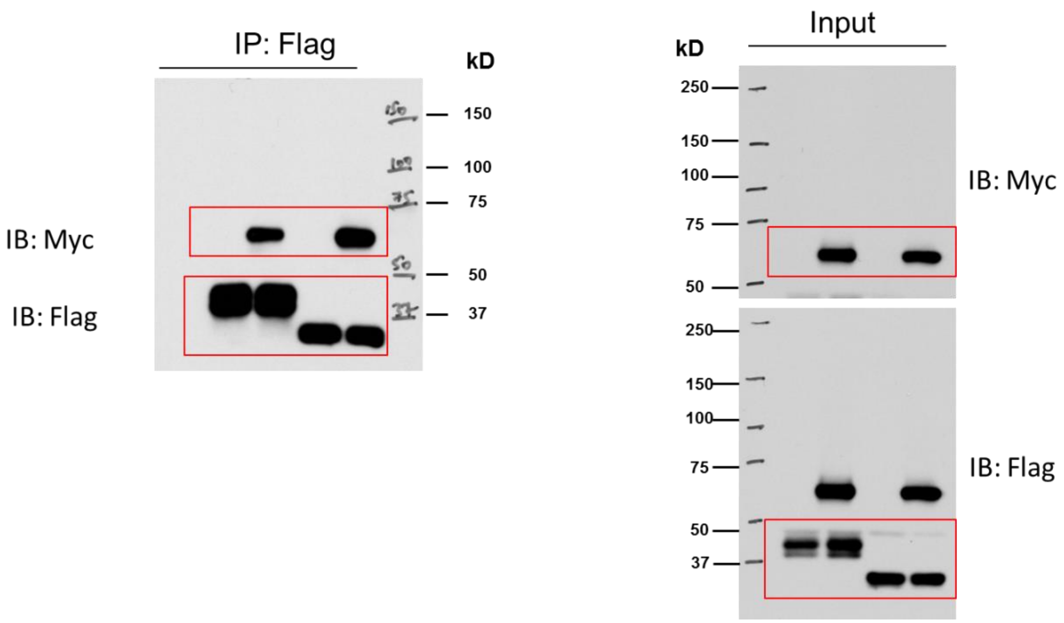


## Supplementary Fig 11

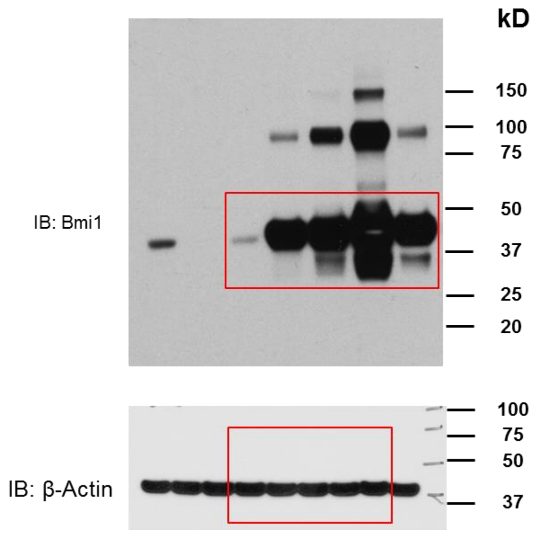

**Supplementary Fig 13**

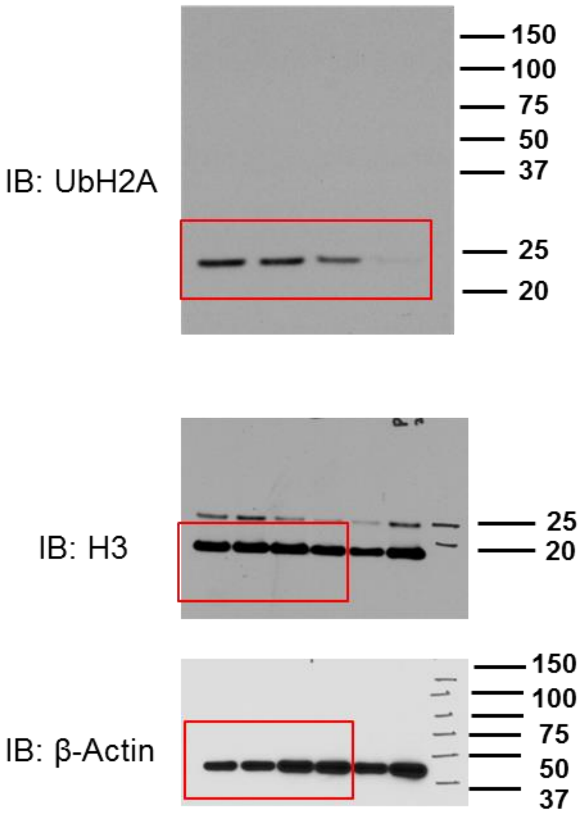


**Supplementary Figure 14.** Uncropped scans of all western blots with reference to original figures. Molecular weight ladder is shown.
